# Supplementary material for: Sorting at embryonic boundaries requires high heterotypic interfacial tension
Source: Nat Commun. 2017 Jul 31;8:157. doi: 10.1038/s41467-017-00146-x (PMC5537356; doi:10.1038/s41467-017-00146-x)
Supplement: Supplementary file 2 — Supplementary Software 1 [file 41467_2017_146_MOESM2_ESM.zip › PottsModel/SrcPottsModel/doc/model/EnergyManager.html]

EnergyManager


JavaScript is disabled on your browser.


Skip navigation links


- Overview
- Package
- Class
- Use
- Tree
- Deprecated
- Index
- Help

- Prev Class
- Next Class

- Frames
- No Frames

- All Classes

- Summary:
- Nested |
- Field |
- Constr |
- Method

- Detail:
- Field |
- Constr |
- Method


model

## Class EnergyManager

- java.lang.Object
- - model.EnergyManager

- ---

    

  ```
  public class EnergyManager
  extends java.lang.Object
  ```

  Manages energy functions.
  Note that this class is writtent such that it is possible to define other energy models.

  Author:
  :   eleyine

- - ### Constructor Summary

    Constructors

    Constructor and Description

    `EnergyManager(Constants c,
    Constants.Model m,
    PottsEngine.StateVariables s)`
  - ### Method Summary

    All Methods Instance Methods Concrete Methods

    | Modifier and Type | Method and Description |
    | --- | --- |
    | `double` | `getDeltaEnergy(Pixel s, Pixel t)` |
    | `java.util.List<EnergyFunction>` | `getEnergyFunctions()` |
    | `java.util.List<EnergyFunction.Energy>` | `getEnergyTypes()` |
    | `Utils.EnergyTracker` | `getTracker()` Makes new tacker based on energy function types. |
    | `void` | `setTracker(Utils.EnergyTracker t)` |

    - ### Methods inherited from class java.lang.Object

      `equals, getClass, hashCode, notify, notifyAll, toString, wait, wait, wait`

- - ### Constructor Detail


    - #### EnergyManager

      ```
      public EnergyManager(Constants c,
                           Constants.Model m,
                           PottsEngine.StateVariables s)
      ```
  - ### Method Detail


    - #### getTracker

      ```
      public Utils.EnergyTracker getTracker()
      ```

      Makes new tacker based on energy function types.


    - #### setTracker

      ```
      public void setTracker(Utils.EnergyTracker t)
      ```


    - #### getEnergyFunctions

      ```
      public java.util.List<EnergyFunction> getEnergyFunctions()
      ```


    - #### getDeltaEnergy

      ```
      public double getDeltaEnergy(Pixel s,
                                   Pixel t)
      ```


    - #### getEnergyTypes

      ```
      public java.util.List<EnergyFunction.Energy> getEnergyTypes()
      ```


Skip navigation links


- Overview
- Package
- Class
- Use
- Tree
- Deprecated
- Index
- Help

- Prev Class
- Next Class

- Frames
- No Frames

- All Classes

- Summary:
- Nested |
- Field |
- Constr |
- Method

- Detail:
- Field |
- Constr |
- Method
